# Placental vascular remodeling in pregnant women with COVID-19

**DOI:** 10.1371/journal.pone.0268591

**Published:** 2022-07-29

**Authors:** Sergiy G. Gychka, Tinatin I. Brelidze, Iurii L. Kuchyn, Tetyana V. Savchuk, Sofia I. Nikolaienko, Volodymyr M. Zhezhera, Ihor I. Chermak, Yuichiro J. Suzuki

**Affiliations:** 1 Department of Pathological Anatomy, Bogomolets National Medical University, Kyiv, Ukraine; 2 Department of Pharmacology and Physiology, Georgetown University Medical Center, Washington, DC, United States of America; 3 Bogomolets National Medical University, Kyiv, Ukraine; 4 OKHMADYT, National Children’s Specialized Hospital, Kyiv, Ukraine; 5 Academy of Human Health, Kyiv City Medical Center, Kyiv, Ukraine; Waseda University: Waseda Daigaku, JAPAN

## Abstract

Severe acute respiratory syndrome coronavirus 2 has been causing the pandemic of coronavirus disease 2019 (COVID-19) that has so far resulted in over 450 million infections and six million deaths. This respiratory virus uses angiotensin-converting enzyme 2 as a receptor to enter host cells and affects various tissues in addition to the lungs. The present study reports that the placental arteries of women who gave birth to live full-term newborns while developing COVID-19 during pregnancy exhibit severe vascular wall thickening and the occlusion of the vascular lumen. A morphometric analysis of the placental arteries stained with hematoxylin and eosin suggests a 2-fold increase in wall thickness and a 5-fold decrease in the lumen area. Placental vascular remodeling was found to occur in all of SARS-CoV-2-positive mothers as defined by RT-PCR. Immunohistochemistry with α-smooth muscle actin and the Kv11.1 channel as well as Masson’s trichrome staining showed that such placental vascular remodeling in COVID-19 is associated with smooth muscle proliferation and fibrosis. Placental vascular remodeling may represent a response mechanism to the clinical problems associated with childbirth in COVID-19 patients.

## Introduction

Coronaviruses are positive-sense, single-stranded RNA viruses that often cause the common cold [1, 2]; however, some coronaviruses can promote severe health problems. Severe acute respiratory syndrome coronavirus 2 (SARS-CoV-2) has been causing the pandemic of coronavirus disease 2019 (COVID-19) [3, 4]. So far, over 450 million people have been infected with SARS-CoV-2, with six million deaths worldwide, causing enormous health, economic, and sociological problems. SARS-CoV-2 enters host cells via the angiotensin-converting enzyme 2 (ACE2) receptor [5, 6]. While lung cells are the primary targets of this respiratory virus, causing acute respiratory distress syndrome [7, 8], SARS-CoV-2 also affects other ACE2-expressing tissues including those of the cardiovascular system [4, 9, 10].

The vascular system is severely affected by COVID-19. We previously reported that the postmortem lung tissues from patients who died of COVID-19-associated acute respiratory distress syndrome (ARDS) exhibited increased vascular wall thickness and reduced lumen compared with those who died of ARDS due to H1N1 influenza infection [11].

COVID-19 is associated with increased neonatal and maternal morbidity and mortality [12–14]. However, the mechanism of the effects of COVID-19 on pregnant women is not understood. Such information should help develop therapeutic strategies to reduce the mortality and morbidity associated with COVID-19.

The present study demonstrates that women who became infected with SARS-CoV-2 during pregnancy and subsequently recovered exhibited severe vascular remodeling of the placental arteries, as determined by histological examinations. Vascular remodeling is associated with increased smooth muscle cell proliferation and fibrosis. These mechanisms may explain how some fetuses and neonates are affected by COVID-19.

## Materials and methods

### Tissue samples

De-identified placental tissues were collected from 113 patients who gave birth to live full-term newborns in Ukraine and archived. Among them, 85 patients were infected with SARS-CoV-2 and developed COVID-19 symptoms at 28–36 weeks of pregnancy. Archiving of these tissues were approved by the regional committee for medical research ethics in Kyiv, Ukraine and performed under the Declaration of Helsinki of 1975 revised in 2013 or comparable ethical standards. All the patients provided written informed consent. We have obtained permission to study these archived samples in accordance with the scientific cooperation agreement between Bogomolets National Medical University and medical institutions across Ukraine. For comparison purposes, we used archived placental tissues from women who underwent live childbirth in 2018 before the COVID-19 pandemic as controls. The ages of mothers, weeks of gestation when the childbirth occurred, placenta weights, and Apgar scores are summarized in Table 1. COVID-19 is defined by the RT-PCR test for SARS-CoV-2. Among the patients in the COVID-19 group, 68% only had mild disease while 32% were diagnosed with severe disease accompanied by pneumonia. Our study groups did not include cases of preeclampsia, hypertension, diabetes, large fetus (>5kg), smoking during pregnancy, or HIV infection. Placentas 5–8 hours after the delivery were fixed overnight in 10% buffered formalin solution (pH 7.4) at room temperature.

**Table 1:** Characteristics of samples used in this study (means ± SD).

	Control (without COVID-19)	With COVID-19
	(N = 105)	(N = 126)
Ages of mothers (years)	30.2 ± 4.7	31.0 ± 4.9
Weeks of gestation (weeks)	39.7 ± 1.7	39.6 ± 4.9
Weight of placenta (grams)	523.1 ± 104.3	543.6 ± 131.1
Apgar	7.9 ± 0.4	7.7 ± 0.8

### Histological examinations

Fixed tissues were embedded in paraffin. From paraffin blocks, 5 μm thick sections were made using a Leica SM 2000 R microtome. The sections were stained with hematoxylin and eosin (H&E) and Masson’s trichrome, and immunohistochemistry using the antibody against α-smooth muscle actin and Kv11.1 channel (Alomone Labs, Jerusalem, Israel) was carried out. Histological specimens were examined using a Leica BX 51 microscope, a Leica MC 190 digital camera, and the Leica LAS software at a magnification of x200. Five photographs were taken for each placenta sample and representative images were selected randomly. Morphometric investigations included the assessment of placental arterial wall thickness and the placental arterial lumen index (the ratio of the internal vessel area to the external vessel area) using the ImageJ software. All the sample preparations were randomized, and the data analyses were performed in a blinded manner.

### Statistical analysis

For the morphometric analysis, IBM SPSS Statistics software version 22.0 was used for the statistical calculations. A Mann-Whitney U test was used to define statistical significance at *p* < 0.05.

## Results

**Fig 1:**
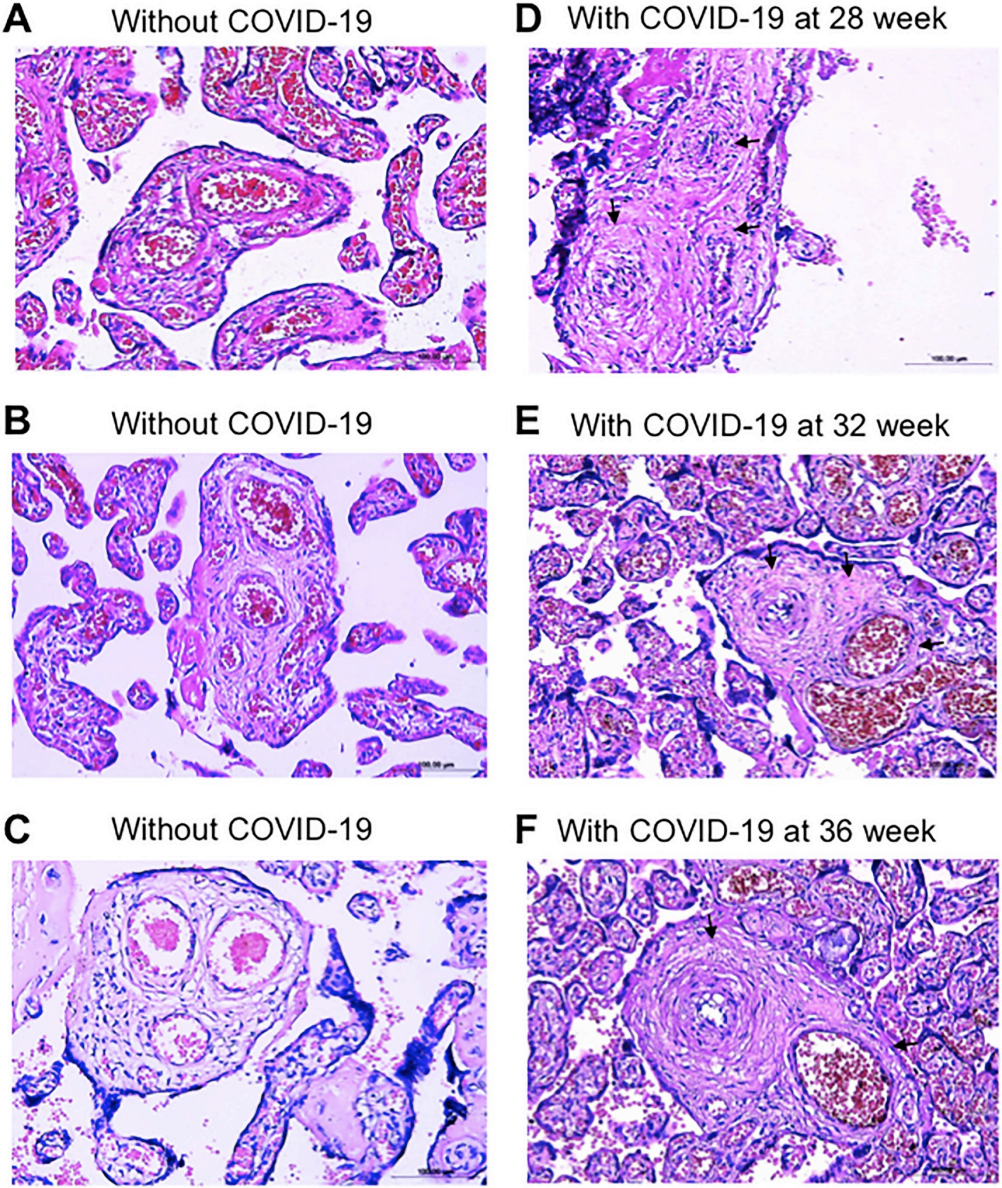
Vascular wall thickening in the placentas of women who contracted COVID-19 during pregnancy. (A-C) H&E staining images of the placental semi-stem villi arteries of three representative women who gave birth without contracting COVID-19 (40 week of gestation). (D-E) H&E staining images of the placental semi-stem villi arteries of three representative women who gave birth (40 weeks of gestation) while contracting COVID-19 at 28, 32, or 36 weeks of gestation. Magnification x200. Arrows indicate remodeled vessels.

Fig 1 shows the representative placental arteries of women who gave birth to live full-term newborns. Panels A-C are the H&E images of the placental arteries of three representative women who gave birth without developing COVID-19. Panels D-F show the H&E images of the placental arteries of three representative women who gave birth but were found to be positive for SARS-CoV-2 at 28, 32, and 36 weeks of pregnancy. The placental arteries of the women who were positive for COVID-19 during pregnancy as determined by RT-PCR tests consistently exhibited histological characteristics of vascular remodeling such as vascular wall thickening and lumen narrowing. By contrast, the placental vessels of the mothers who were free from the SARS-CoV-2 infection during pregnancy showed no occurrences of thickened placental arteries.

**Fig 2:**
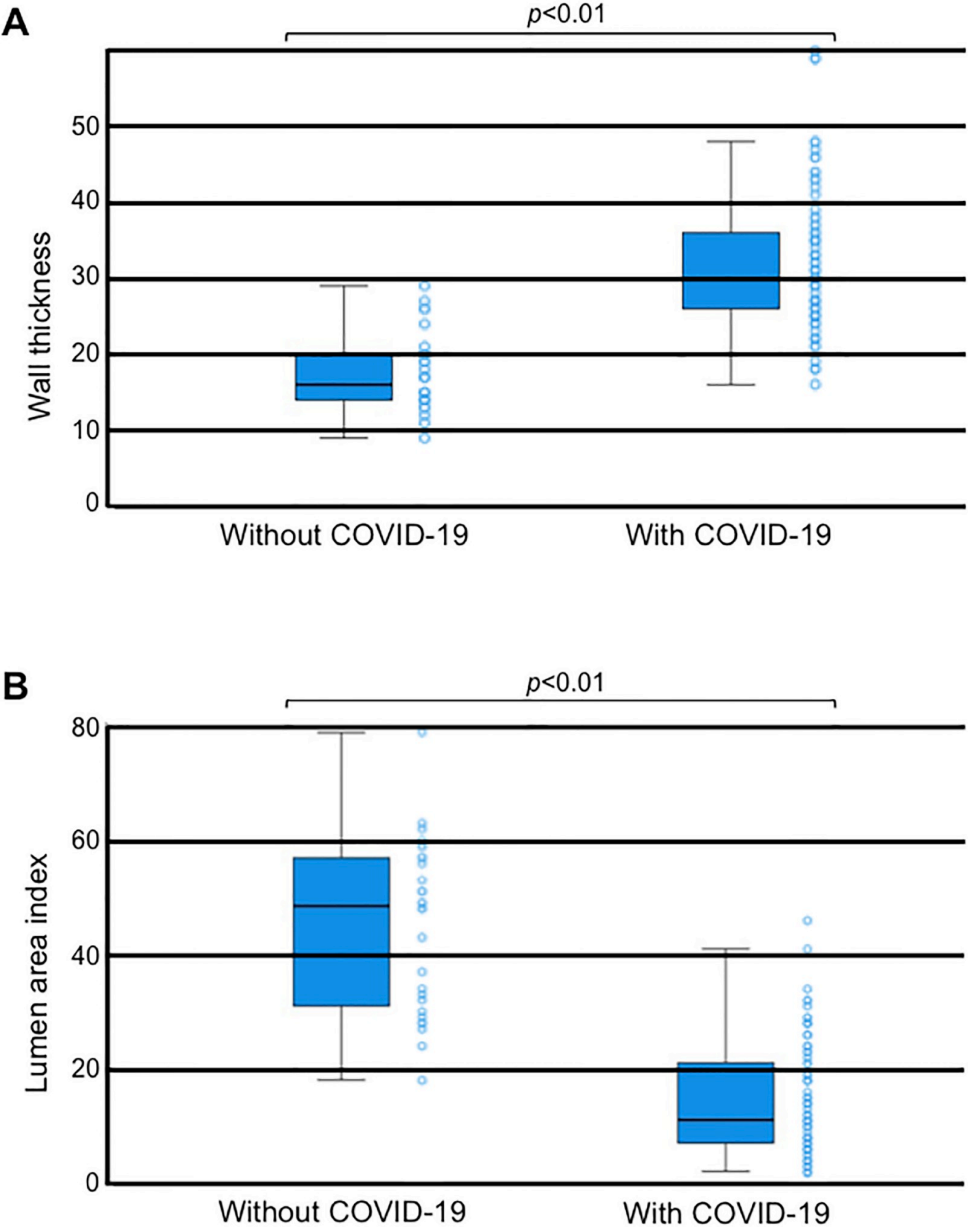
Morphometric analysis of the placental arterial wall thickness in women who gave birth without or with COVID-19. Box plots represent (A) vascular wall thickness and (B) the lumen area index values. N = 28 for control and N = 85 for COVID-19. Mann-Whitney U test indicated that the two values are significantly different at *p <* 0.01.

The morphometric analysis of placental arterial wall thickness showed that the median value for COVID-19 patients was ~30 μm (N = 85 placentas), while that for controls was ~15 μm (N = 28 placentas), indicating that placental arterial walls are twice as thick in COVID-19 patients than in those without COVID-19 (Fig 2A). These two values are significantly different (*p* < 0.01). The placental arterial lumen area was found to be significantly smaller (5-fold) in COVID-19 patients than in controls (*p* < 0.01, Fig 2B).

**Fig 3:**
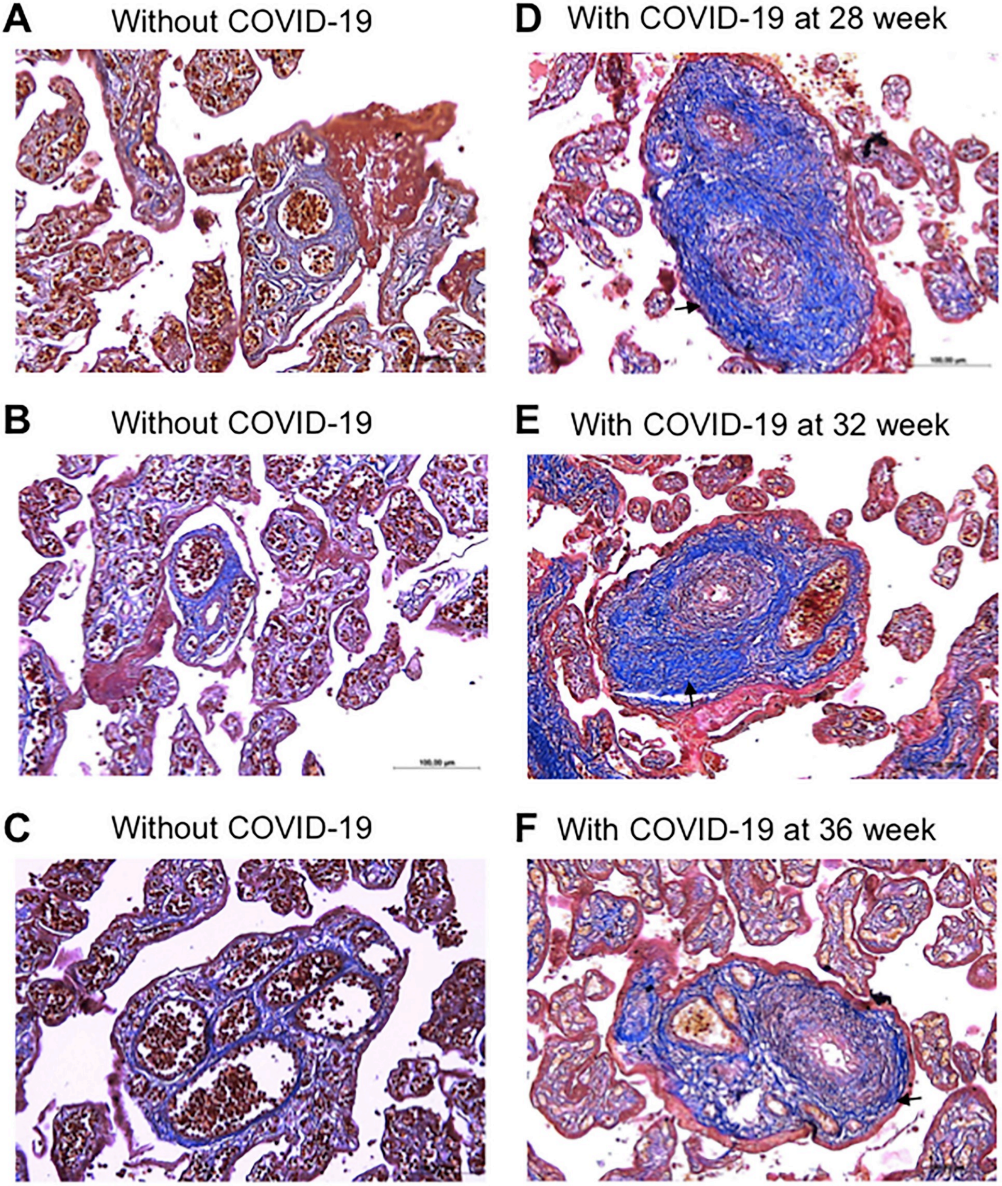
Masson’s trichrome staining. (A-C) Placentas of three representative women who gave birth without contracting COVID-19 (40 week of gestation). (D-E) Placentas of three representative women who gave birth (40 weeks of gestation) while contracting COVID-19 at 28, 32, or 36 weeks of gestation. Magnification x200. Arrows indicate remodeled vessels.

As shown in Fig 3, Masson’s trichrome staining showed that women who contracted SARS-CoV-2 during pregnancy and had quantitatively thickened placental vessels as determined in Fig 2 exhibited fibrosis of the vascular walls and perivascular space in semi-stem placenta villi.

**Fig 4:**
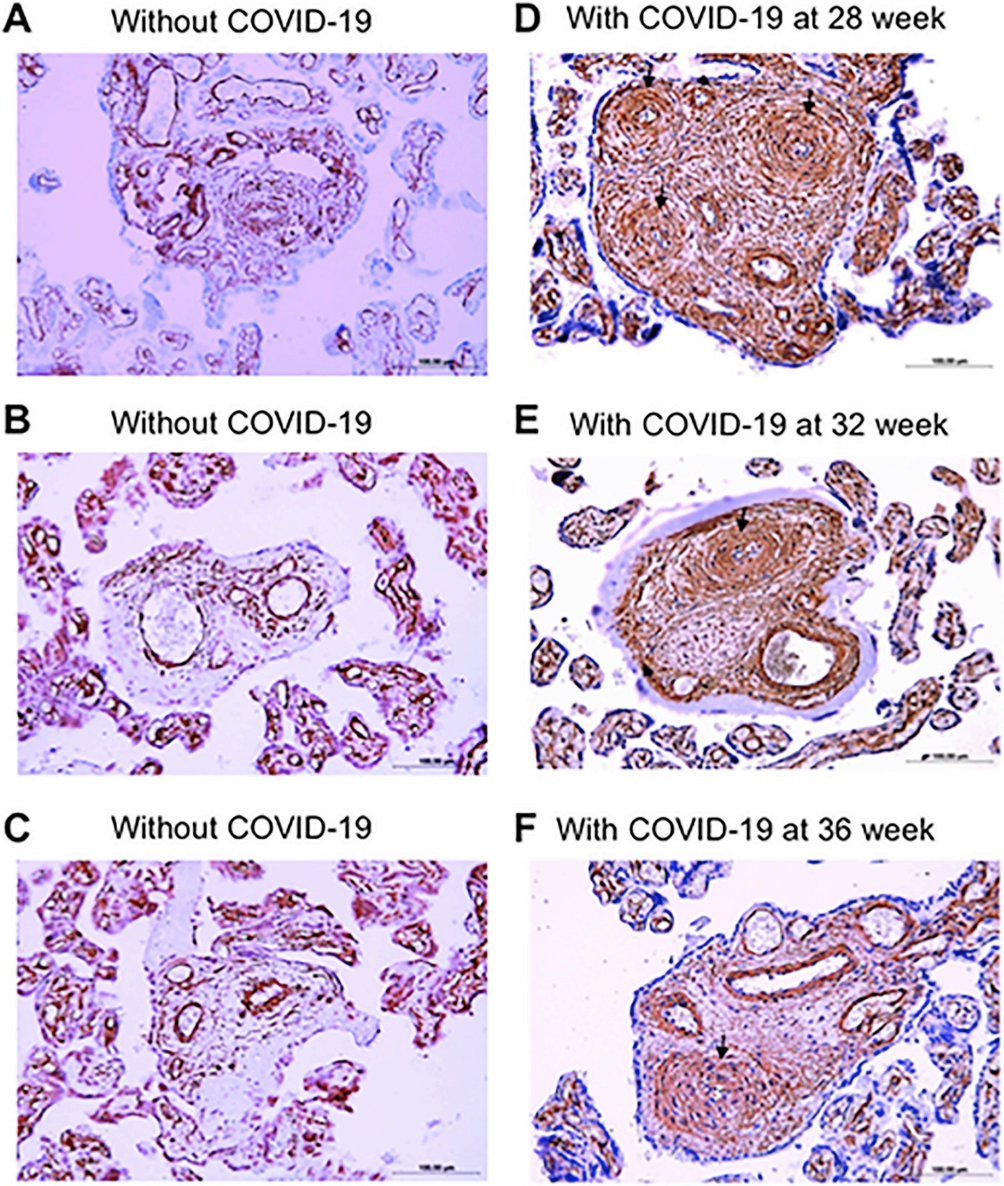
Immunohistochemistry of α-smooth muscle actin in the placental arteries of women who gave birth without or with COVID-19 during pregnancy. (A-C) Immunohistochemistry images of three women who gave birth without contracting COVID-19 (40 weeks of gestation). (D-F) Immunohistochemistry images of three women who gave birth (at 40 weeks of gestation) but contracted COVID-19 at 28, 32, or 36 weeks of gestation. Magnification x200. Arrows indicate remodeled vessels.

Immunohistochemistry using a smooth muscle cell marker, α-smooth muscle actin, clearly indicated the dramatic increase in smooth muscle mass in the placental arteries of COVID-19 patients with quantitatively thickened placental vessels as determined in Fig 2 (panels D-F, Fig 4) compared with controls without COVID-19 (panels A-C, Fig 4). Given the substantial increase in the mass of smooth muscle in the medial layer (Figs 2 and 4), it is likely that the placental arteries of COVID-19 patients underwent smooth muscle cell proliferation.

**Fig 5:**
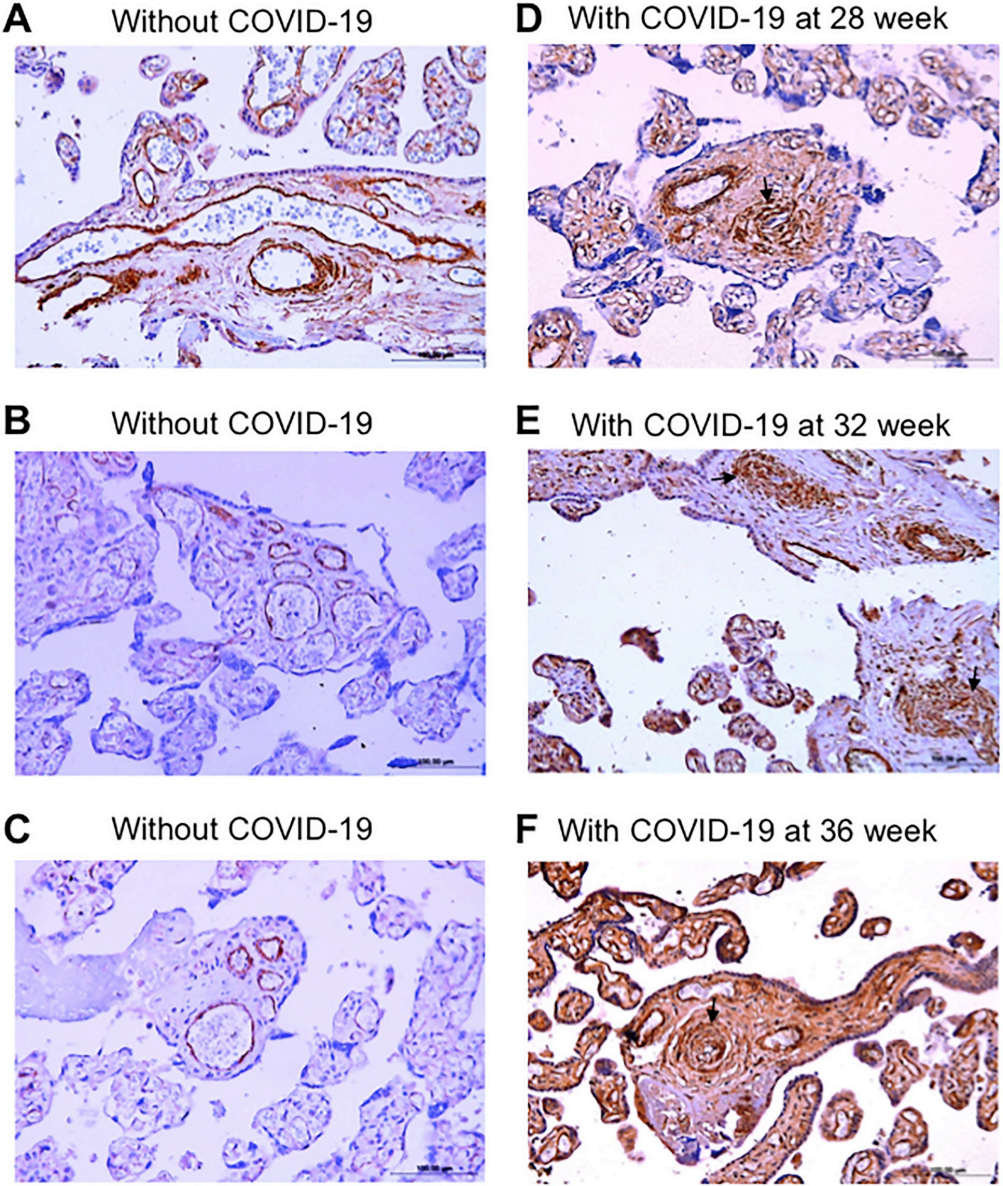
Immunohistochemistry of the Kv11.1 channels in the placental arteries of women who gave birth without or with COVID-19 during pregnancy. (A-C) Immunohistochemistry images of three women who gave birth without contracting COVID-19 (40 weeks of gestation). (D-F) Immunohistochemistry images of three women who gave birth (40 weeks of gestation), but contracted COVID-19 at 28, 32, or 36 weeks of gestation. Magnification x200. Arrows indicate remodeled vessels.

We previously found that lung vascular smooth muscle cells express Kv11.1 potassium channels and that the expression of these channels increases in association with vascular remodeling in patients with chronic obstructive pulmonary disease [15]. The remodeled placental vessels of SARS-CoV-2-infected mothers also exhibited increased Kv11.1 expression (Fig 5).

## Discussion

The major finding of this study is that the placental arteries of women who gave birth to live full-term newborns, but contracted SARS-CoV-2 during pregnancy, exhibited severe vascular remodeling. Such changes in placental vessels occurred in all mothers who tested positive for COVID-19 as determined by RT-PCR tests, irrespective of the presence or absence of COVID-19-associated symptoms.

The narrowing of the placental arterial lumen is expected to alter the blood flow between the mother and fetus. In the population investigated in this study, despite the occurrence of severe placental vascular remodeling, COVID-19 did not significantly impact the health of the newborn children according to the evaluations of appearance, pulse, grimace, activity, and respiration, as quantified by Apgar scores. However, in our participating hospitals in Ukraine, among the group of 414 women infected with SARS-CoV-2 during pregnancy, 20 cases of antenatal fetal death were reported at a rate of almost 5%, which is vastly above the population average. Further work is thus needed to determine the effect of COVID-19 on pregnancy as well as the effects of placental vascular remodeling on neonatal well-being and development.

Cardiovascular diseases are linked to the development of severe and possibly fatal conditions of COVID-19 [4, 9, 10]. Thus, the pathology of COVID-19 does not seem to be explained merely by the action of SARS-CoV-2 entering host cells for destruction. Previous studies of the lung vasculature have proposed that SARS-CoV-2 spike protein-mediated cell signaling promotes the hyperplasia and/or hypertrophy of vascular smooth muscle and endothelial cells [11]. More recently, *in vivo* animal studies have confirmed that the spike protein (without the rest of SARS-CoV-2) can adversely influence the vascular system [16]. Because our results indicated that placental vascular remodeling occurred in all the pregnant women who become positive for SARS-CoV-2, even though they did not develop severe COVID-19, the spike protein may play a role in the mechanism. Further studies investigating the mechanism of placental vascular remodeling would be invaluable to develop effective therapeutic strategies to reduce the mortality and morbidity associated with COVID-19.

We previously reported that lung vascular smooth muscle cells express Kv11.1 potassium channels and that their expression increases with the increased vascular remodeling in patients with chronic obstructive pulmonary disease [15]. Similar results were observed in a rat model of pulmonary arterial hypertension, showing that a Kv11.1 channel inhibitor prevented the vascular remodeling associated with pulmonary arterial hypertension [15]. Here, we report that placental vessels of SARS-CoV-2-infected mothers also exhibited increased Kv11.1 expression. Thus, the role of the Kv11.1 channel and therapeutic potential of its inhibitors should also be explored in COVID-19-associated placental vascular remodeling in pregnant mothers.

In summary, the present study found that placental vascular walls thickened and the lumen narrowed in women who gave live birth but contracted COVID-19 during pregnancy. Smooth muscle proliferation and collagen fiber deposition appear to play major roles in the development of placental vascular remodeling. Elucidating the mechanism of these events should help provide new therapeutic targets to combat COVID-19, and prevent stillbirth and premature birth, as well as alterations of child development.
